# Low levels of amyloid-beta and its transporters in neonatal rats with and without hydrocephalus

**DOI:** 10.1186/1743-8454-6-4

**Published:** 2009-05-26

**Authors:** Kelley E Deren, Jennifer Forsyth, Osama Abdullah, Edward W Hsu, Petra M Klinge, Gerald D Silverberg, Conrad E Johanson, James P McAllister

**Affiliations:** 1 Department of Neurosurgery, Division of Pediatric Neurosurgery, Primary Children's Medical Center and the University of Utah, Salt Lake City, Utah 84132, USA; 2 Department of Bioengineering and Brain Imaging Center University of Utah, Salt Lake City, Utah 84112, USA; 3 International Neuroscience Institute Hannover and the Neurosurgical Department, Hannover, D-30625, Germany; 4 Department of Clinical Neuroscience, Aldrich Laboratories, Rhode Island Hospital, Warren Alpert Medical School, and Brown University Providence, Rhode Island 02903, USA; 5 Department of Neurosurgery, Rhode Island Hospital and Brown University Providence, Rhode Island 02903, USA

## Abstract

**Background:**

Previous studies in aging animals have shown that amyloid-beta protein (Aβ) accumulates and its transporters, low-density lipoprotein receptor-related protein-1 (LRP-1) and the receptor for advanced glycation end products (RAGE) are impaired during hydrocephalus. Furthermore, correlations between astrocytes and Aβ have been found in human cases of normal pressure hydrocephalus (NPH) and Alzheimer's disease (AD). Because hydrocephalus occurs frequently in children, we evaluated the expression of Aβ and its transporters and reactive astrocytosis in animals with neonatal hydrocephalus.

**Methods:**

Hydrocephalus was induced in neonatal rats by intracisternal kaolin injections on post-natal day one, and severe ventriculomegaly developed over a three week period. MRI was performed on post-kaolin days 10 and 21 to document ventriculomegaly. Animals were sacrificed on post-kaolin day 21. For an age-related comparison, tissue was used from previous studies when hydrocephalus was induced in a group of adult animals at either 6 months or 12 months of age. Tissue was processed for immunohistochemistry to visualize LRP-1, RAGE, Aβ, and glial fibrillary acidic protein (GFAP) and with quantitative real time reverse transcriptase polymerase chain reaction (qRT-PCR) to quantify expression of LRP-1, RAGE, and GFAP.

**Results:**

When 21-day post-kaolin neonatal hydrocephalic animals were compared to adult (6–12 month old) hydrocephalic animals, immunohistochemistry demonstrated levels of Aβ, RAGE, and LRP-1 that were substantially lower in the younger animals; in contrast, GFAP levels were elevated in both young and old hydrocephalic animals. When the neonatal hydrocephalic animals were compared to age-matched controls, qRT-PCR demonstrated no significant changes in Aβ, LRP-1 and RAGE. However, immunohistochemistry showed very small increases or decreases in individual proteins. Furthermore, qRT-PCR indicated statistically significant increases in GFAP.

**Conclusion:**

Neonatal rats with and without hydrocephalus had low expression of Aβ and its transporters when compared to adult rats with hydrocephalus. No statistical differences were observed in Aβ and its transporters between the control and hydrocephalic neonatal animals.

## Background

Hydrocephalus is a condition characterized by an excessive build up of cerebrospinal fluid (CSF) in the cerebral ventricles. This condition is most frequently found in children but also occurs in aged adults. Practically all forms of hydrocephalus exhibit impairment in CSF flow and absorption. This occurs in extraventricular (communicating) hydrocephalus, for example, when meningitis causes fibrosis and reduces CSF flow through the subarachnoid space[1]; it occurs in intraventricular (obstructive) hydrocephalus when a tumor or congenital dysplasia physically blocks any portion of the cerebral ventricles (often the most narrow regions such as the cerebral aqueduct of Sylvius) causing the proximal parts of the ventricular system to dilate. In any case, the resulting reduced CSF flow diminishes the natural CSF function of maintaining homeostasis and leads to various neurological deficits [2-7]. Interruption in normal CSF flow pathways create a deficiency in the ability of the CSF to clear toxic substances from the brain[8,9]. In particular, one important substance affected by impaired clearance is amyloid-beta protein (Aβ). Accumulation of Aβ is associated with Alzheimer-type dementia and is commonly found in the CSF of adults with normal pressure hydrocephalus (NPH) and in brain tissue from adult rat models of this disorder. [7,10-16].

Although the prevalence of neonatal hydrocephalus is relatively high, no studies have examined protein clearance mechanisms in children with hydrocephalus or immature experimental animals with this disorder. Based on the findings in the adult literature, we hypothesized that impaired clearance of Aβ also occurs in the neonatal hydrocephalic brain and is accompanied by alterations in Aβ transporters. The low-density lipoprotein receptor-related protein-1 (LRP-1) is believed to be the major transporter of Aβ from the brain parenchyma into the bloodstream, while the receptor for advanced glycation end products (RAGE) delivers Aβ into the brain from the vasculature[14]. Furthermore, adult studies have shown a correlation between astrocytes and Aβ in cases of NPH and AD [17-26]. While GFAP labeled astrocytes are commonly increased in hydrocephalus of all ages, probably in response to a wide variety of injury mechanisms (reviewed in[4,5,19,27,28]), these astrocytes could also be related to Aβ clearance. For that reason, we hypothesized that there would be an association between Aβ and astrocytes with the progression of hydrocephalus.

The present study has examined LRP-1, RAGE, Aβ, and GFAP in neonatal animals after intraventricular (obstructive) hydrocephalus was induced by kaolin injections into the cisterna magna at 1 day of age and ventriculomegaly had progressed until 21 days of age.

## Methods

### Animals

Time-pregnant Sprague Dawley rats were purchased from Charles River, (Wilmington, MA, USA) and delivered to the University of Utah on the 19^th ^day of gestation. The dam and litter were housed on a 12-h light/dark cycle throughout the protocol. At birth (approximately 21 days of gestation) pups were divided into two groups: sham control (n = 10), and hydrocephalic (n = 10). Five animals from each group were analyzed by immunohistochemistry and the other five animals in each group were analyzed by qRT-PCR. All pups remained with the dam until sacrifice on post-kaolin day 21. In addition, tissues from adult rats for age-related comparisons were obtained from previous studies, i.e. 6 month old kaolin injected rats[29] and 12 month old kaolin-injected rats[15]. All animal experiments were conducted in accordance with the guidelines of the NIH Care and Use of Laboratory Animals and approved by the University of Utah (neonatal animals), Wayne State University (6 month animals), and the International Neuroscience Institute, Hannover, Germany (12 month animals). All efforts were made to minimize discomfort and the number of animals used.

### Surgical induction of obstructive hydrocephalus

Kaolin induction of hydrocephalus was performed on post-natal day one using aseptic techniques as described previously[30]. Anesthesia was achieved by hypothermia on a bed of ice since newborn rats have no thermoregulatory ability. Once the animal reached a surgical plane of anesthesia (loss of knee-jerk reflexes), the dorsal surface of the skull and the cervical musculature were exposed through a midline incision that extended from the center of the skull to the end of the second cervical vertebra. The scalp, membranous soft tissue, and cervical musculature were reflected, exposing the occipital bone, the dorsal portion of the C1 vertebra, and the atlanto-occipital membrane which covers the 1 mm interval between those two bones. A 30-gauge needle attached to a 1.0 ml syringe containing 25% kaolin in sterile saline was inserted through the atlanto-occipital membrane and the dura mater into the cisterna magna. CSF was allowed to escape and thereby compensated for the injected volume of kaolin. Approximately 25 μl of kaolin was administered at a rate of 6 μl/sec. Following the injection, the underlying muscles were closed with 7-0 absorbable suture and the skin closed with 7-0 Prolene suture. The animal was taken off the ice bath and slowly warmed. Once the pup became active and responsive, exhibiting normal knee jerk reflexes, it was returned to the dam and litter. Sham control animals underwent identical procedures used for kaolin induction, but sterile saline was injected instead of kaolin.

### Magnetic resonance imaging

On post-injection day 10, magnetic resonance imaging (MRI) using T2-weighted images was employed to confirm or rule out ventriculomegaly. The sham injected and kaolin injected animals were anesthetized with 1–3% isoflurane through a ventilator which included self-excavating components (Harvard Apparatus part # 723011 and 723026, Holliston, Massachusetts, USA) and placed in the prone position on a cradle with gauze padding around the pup for warmth while in the magnet. All measurements were performed on a 7.0-Tesla horizontal-bore animal MRI scanner (Bruker Biospec, Billerica, Massachusetts, USA) with a 12-cm-bore actively shielded gradient coil set capable of producing magnetic field gradients of up to 600 mT/m. For homogeneous radiofrequency (RF) excitation, a 7.2-cm-inner-diameter whole-body birdcage RF coil and a 3.0 cm-diameter surface coil were respectively used for transmission and reception, with active RF decoupling to avoid signal interference. Once the pups regained consciousness they were returned to the dam. On post-kaolin day 21, a second MRI was performed prior to sacrifice for ventricular size comparison, using the procedures described previously.

Ventriculomegaly was quantified from the MRI images using the Evan's ratio. This measurement is used routinely in clinical practice and is calculated from coronal slices as the ventricular width divided by the brain width at the level of the foramina of Monro. Statistical comparisons between control and hydrocephalic groups were performed with Student's t-test.

### Immunohistochemistry

On post-injection day 21 immediately following the MRI, 5 animals from each neonatal group (saline control and hydrocephalic) to be analyzed by immunohistochemistry were anesthetized with Nembutal (120 mg/kg, IP) and perfused transcardially with 4% buffered paraformaldehyde. Prior to paraffin embedding, hydrocephalic brains were injected with a solution of 4% agar in dH_2_O in order to provide support to the thinned and fragile cortical mantle, as described previously[27]. The tissue was embedded according to routine protocols, sectioned in the coronal plane at a thickness of 15 μm, and sections mounted at 20 μm intervals on glass slides.

All slides were rehydrated prior to immunohistochemical staining. Tissue sections for immunohistochemistry staining of RAGE, LRP-1, and GFAP underwent antigen retrieval by placing slides in a steamer for 20 min with 10 mM citrate buffer (pH 6.0), preheated to 90–100°C. The slides were cooled for 20 min in the buffer and washed in 0.01 M PBS. Following antigen retrieval, the tissue sections were quenched with 3% H_2_O_2 _for 20 min at room temperature to eliminate endogenous peroxidase activity and then washed with distilled water.

For RAGE staining, serum-free protein blocker was added for 5 min and drained. Primary RAGE antibody (Affinity Bioreagents, Golden, Colorado, USA) was applied at a dilution of 1:100 at room temperature for 2 h and then washed with PBS. Secondary anti-rabbit IgG biotinylated antibody (1:200, Vector Laboratories, Burlingame, California, USA) was added for 45 min at room temperature and then rinsed with PBS. For primary antibody detection, sections were treated with avidin-biotin complex (ABC Kit, Vector Laboratories) for 30 min, and then rinsed with PBS. Lastly, the sections were developed with diaminobenzidine (DAB Kit, Vector Laboratories) for 5 min and rinsed with PBS. Sections were counterstained with cresyl violet. Staining for LRP-1 (1:100, Orbigen San Diego, CA, USA) and GFAP (1:500, Dako Cytomation, Glostrup, Denmark) followed the same procedures as for RAGE.

Tissue sections for Aβ_(1–42) _staining underwent antigen retrieval with 10% formic acid at a pH of 1.8 for 20 min at room temperature. Sections were rinsed with distilled water and quenched with 3% H_2_O_2 _for 20 min at room temperature and rinsed again with distilled water. 1% normal goat serum was used as a blocking agent for 5 min and drained. Primary Aβ_(1–42) _antibody (1:100, Affinity Bioreagents) was applied for 2 h at room temperature and washed with PBS. Secondary anti-rabbit IgG biotinylated antibody (1:200, Vector Laboratories) was added for 1 h at room temperature and washed with PBS. Following the secondary antibody, ABC was added for 30 min, washed with PBS, and developed with DAB. Sections were counterstained with cresyl violet.

All tissue was analyzed with light microscopy using bright-field optics. Analysis included qualitative observations of the immunoreactivity of GFAP, and identification of positively-stained RAGE, LRP-1 and Aβ proteins in the cerebral cortex and hippocampus. Subjective judgments were made by two microscopists on the presence or absence of immunolabeling, based on previous reports of the light microscopic appearance of these markers[14,15,27].

### Quantitative real time (qRT)- PCR

On post-injection day 21, animals to be analyzed by qRT-PCR were sacrificed with 120 mg/kg of Nembutal i.p. immediately following MRI. The parietal cortex and the hippocampus were dissected, placed into individual micro-centrifuge tubes, and immediately frozen in liquid N_2_. Tissue was stored at -80°C until needed for analysis.

Total RNA was extracted from frozen parietal cortex and hippocampal tissue using the Nucleospin RNA II mini columns (Clontech, Mountain View, CA, USA). Samples were DNAse treated according to manufacturer's specifications to ensure removal of DNA. RNA quality and concentration were analyzed using the Thermo Scientific NanoDrop™ 1000 Spectrophotometer (Thermo Fisher Scientific, Waltham, Massachusetts, USA) and subsequently reverse transcribed using the SuperScript First-Strand Synthesis System for qRT-PCR (Invitrogen, Carlsbad, California, USA) as instructed by the supplier. Real-time PCR was performed with each sample in replicates of four with the ABI Prism 7900HT Sequence Detection System using TaqMan Gene Expression Assays for LRP-1 (Rn01160459_g1), RAGE (Rn00584249_m1), and GFAP (Rn00566603_m1) (Applied Biosystems, Foster City, California, USA). Glyceraldehyde-3-phosphate dehydrogenase (Rn99999916_s1) was used as a reference gene.

Relative expression levels were determined using the comparative Ct method as used in previous studies[31]. Briefly, values were presented as 2^-(Δ Ct) ^where the Δ Ct value equals the Ct of the gene of interest minus the Ct of the internal control. Results were regarded as statistically significant with a 95% confidence interval (*P *< 0.05) using the Student's t test.

## Results

### MRI imaging

**Figure 1:**
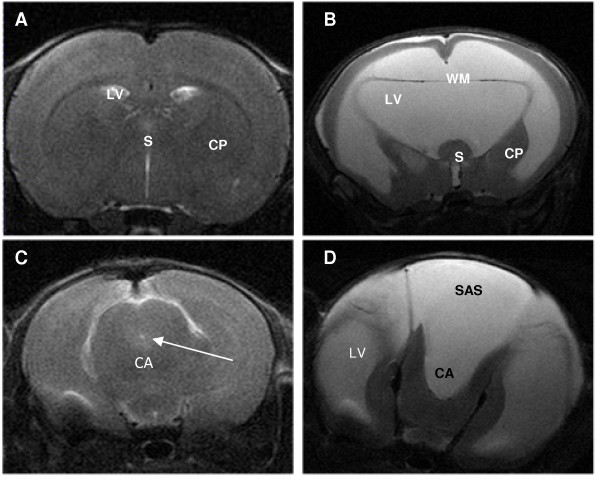
**Representative coronal slices of T2-weighted MRI images comparing 21 day post-saline controls (A, C) to 21 day post-kaolin hydrocephalic brains (B, D)**. The hydrocephalic brains exhibited a dramatic increase in size of the lateral ventricles (LV), as well as the gross distortion of the caudate putamen (CP) and septum (S) following kaolin injections. The periventricular white matter (WM) appears to be thinned, stretched, and detached from the overlying cortical grey matter by either expansion of the lateral ventricle or edematous tissue. Additionally, image D demonstrates the cerebral aqueduct (CA) expanding caudally creating a possible communication to the subarachnoid space (SAS) that overlies the dorsal surface of the midbrain.

**Figure 2:**
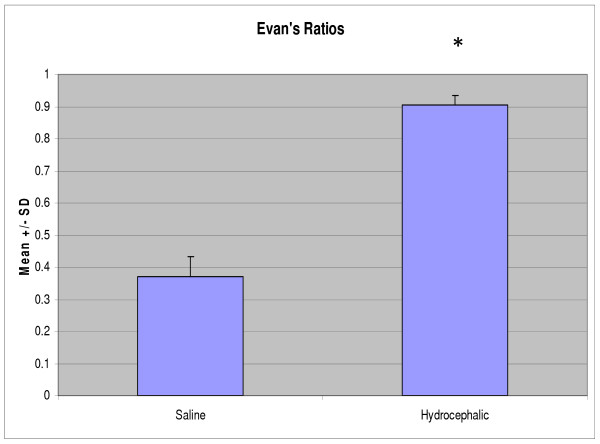
**Graph representing ventricular size, expressed as the mean Evan's ratios of the two groups**. The ventricles of the hydrocephalic group (n = 5) were significantly increased in size (*, *P *< 0.001) when compared to the saline controls (n = 5).

No animals that received saline injections into the cisterna magna developed ventriculomegaly (Fig. 1A). Animals that received kaolin injections into the cisterna magna developed hydrocephalus within a few days which was severe by 21 days (Fig 1B). Grossly, the lateral ventricles enlarged more than the third ventricle, which enlarged more than the fourth ventricle. The septum pellucidum was also obliterated and there was a large communication across the midline between the two lateral ventricles. The cerebral aqueduct was normal in the saline controls (Fig 1C) but in the hydrocephalic animals was expanded caudally creating a possible communication to the subarachnoid space (SAS) that overlies the dorsal surface of the midbrain (Fig 1D). The expansion of the cerebral ventricles was reflected in the Evan's ratios. Student's t-test showed a significant increase (*P *< 0.001) when compared to the control group (Fig. 2).

### Immunohistochemistry of amyloid-β protein and transporters

**Figure 3:**
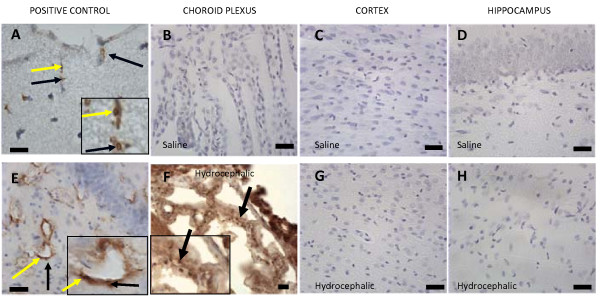
**Photomicrographs representing amyloid beta immunohistochemistry of an adult control animal that is positively stained with Aβ antibody(positive control; A, E), 21-day saline controls (B, C, D), and age-matched hydrocephalic animals (F, G, H)**. In the adult tissue at higher magnification (A, E), positive Aβ labeling can be seen as punctate or more diffuse profiles within and adjacent to the walls of capillaries. In the choroid plexus of neonatal animals, positive Aβ staining, characterized by dark brown punctate particles (arrows) in individual cells, was only seen in hydrocephalic animals. In the cortex and hippocampus, no Aβ labeling was found in either 21-day hydrocephalic animals (G, H) or age-matched controls (C, D). In A, E, and F, the inset represents a higher magnification to show the punctate character of some Aβ labeling. Scale bar = 25 μm.

LRP-1, RAGE, and Aβ were qualitatively evaluated based on the presence of their respective antibody reactions in three different areas: choroid plexus, cerebral cortex, and hippocampus, where those proteins have been previously shown to be located in aged adults with NPH and aged rats with hydrocephalus[13-15,32]. Aβ immunostaining (Fig. 3) was present in the choroid plexus of the 21-day hydrocephalic animals where the positive labeling was prominent in the epithelial cells. This was not seen the choroid plexus of saline controls. No trace of Aβ could be found in the neurons or around the blood vessels of the cerebral cortices and hippocampi in 21-day normal control or age-matched hydrocephalic animals.

**Figure 4:**
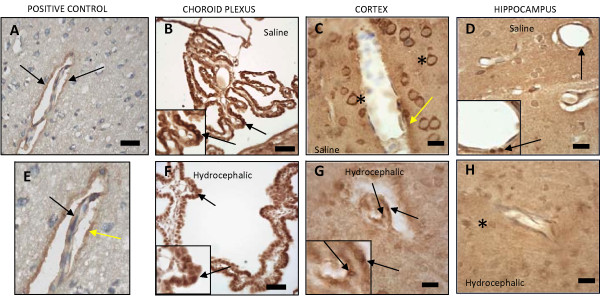
**Photomicrographs representing LRP-1 immunohistochemistry of an adult control animal that is positively stained with LRP-1 (positive control; A, E), 21-day saline controls (B, C, D), and age-matched hydrocephalic animals (F, G, H)**. Black arrows represent blood vessels that are positively stained with LRP-1 proteins. These are characterized by either dark brown punctate particles (D, G) or diffused profiles (A, B, C, F). The asterisks represent non-specific staining of neuron and glial cell bodies. At higher magnification (E) positive LRP-1 labeling can be seen both within the endothelial lining (black arrow) of a capillary deep within the cerebral cortex, as well as in the neuropil immediately adjacent (yellow arrow) to the microvessel. In the choroid plexus of 21-day controls (B), LRP-1 labeling appears throughout the cytoplasm of epithelial cells but seems more intense on the apical surface adjacent to CSF. In 21-day hydrocephalic animals (F), LRP-1 labeling is also present throughout the cell but seems denser in central regions of the cell, which may mean that LRP-1 has translocated to the nucleus. Insets show these features at higher magnification. There are minimal changes between the hydrocephalic animals (G, H) versus the saline controls (C, D), i.e. only a slight increase in the cortex and a slight decrease in the hippocampus. In D and G, the insets represent a higher magnification to show the punctate character of some LRP-1 labeling. Scale bar = 25 μm.

LRP-1 immunostaining in 21-day normal control and aged-matched hydrocephalic animals (Fig. 4) showed positive labeling occurring in the choroid plexus along the apical surface and throughout the cytoplasm of the epithelial cells in both groups. In the 21-day hydrocephalic animals LRP-1 labeling appears denser in central regions of the cell. In the cerebral cortex, as described in the caption for Figure 4, LRP-1 labeling in hydrocephalic animals was compared to both a normal adult LRP-1-positive control and 21-day saline-injected controls. There were minimal traces of LRP-1 in the cortices of control animals. Although sections exhibited considerable non-specific labeling of neurons and glial cells, labeling occurred in the endothelium of microvessels and in the neuropil adjacent to these structures. In the hydrocephalic animals, there was a slight increase in LRP-1 labeling in the vasculature of layer IV. In contrast to the cortex, the hippocampus of hydrocephalic animals exhibited slight decreases in LRP-1 labeling. The blood vessels along the hippocampal fissure and in the pyramidal layer of the hippocampus in the control animals were positively stained with LRP-1; none of this labeling was found in the hydrocephalic group.

**Figure 5:**
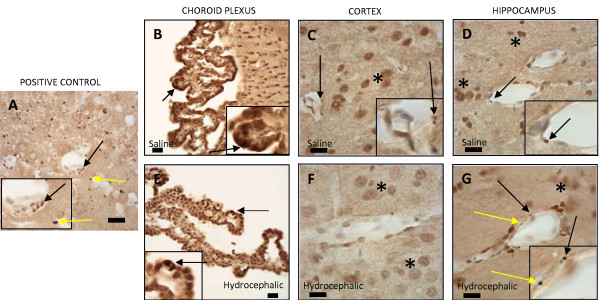
**Photomicrographs representing RAGE immunohistochemistry of a positive control (adult control animal that is positively stained with RAGE; A), 21-day saline controls (B, C, D), and age-matched hydrocephalic animals (E, F, G)**. Arrows represent blood vessels that are positively stained with RAGE proteins, characterized by either dark brown punctate particles (A, C, D, G) or diffused profiles (B, E). The asterisks represent non-specific staining of neuron and glial cell bodies. At higher magnification (inset), positive RAGE labeling can be seen within the endothelial lining (arrow) of a capillary deep within the hippocampus. In the choroid plexus of 21-day controls (B), RAGE labeling appears throughout the cytoplasm of epithelial cells but seems more intense on the apical surface adjacent to CSF. In 21-day hydrocephalic animals (E), RAGE labeling is also present throughout the cell but seems to have translocated to the nucleus. Insets show these features at higher magnification. There are minimal differences between the hydrocephalic animals (F, G) versus the saline controls (C, D), i.e. only a slight decrease in the cortex and no change in the hippocampus. In C, D and G, the insets represent a higher magnification to show the punctate character of some RAGE labeling. Scale bar = 25 μm for all panels.

Positive antibody staining for RAGE (Fig. 5) appeared along the apical surface and throughout the cytoplasm of the epithelial cells in the choroid plexus of both the normal control and aged-matched hydrocephalic animals. When examining the cerebral cortex of the saline control animals, RAGE immunostaining was found around the blood vessels in layers II, III, and IV, but there was no trace of RAGE in the cortex of the hydrocephalic animals. Moreover, similar to LRP-1, non-specific labeling was found in the neurons and glial cells in the cortices of both animals. The hippocampus in both the 21 day saline controls and the hydrocephalic animals exhibited the presence of RAGE around the microvessels but there was no noticeable difference between the two groups.

### Reactive astrocytosis in neonatal-onset hydrocephalus

**Figure 6:**
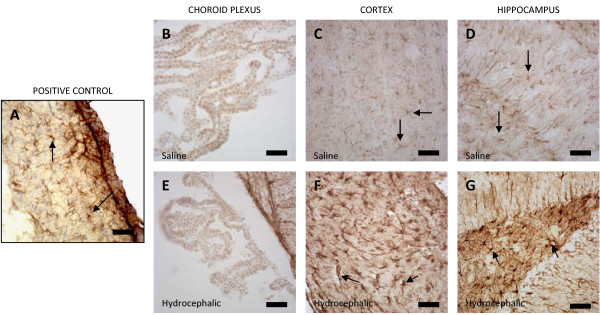
**Representative immunohistochemical images of anti-GFAP staining**. The positive control (A) is provided to illustrate typical staining characteristics. The hydrocephalic cortex and hippocampus contain severe reactive astrocytosis (F and G respectively) when compared to their age-matched saline controls (C and D). The choroid plexus B and E in both groups were negative for GFAP. Arrows = reactive astrocytes, scale bar = 25 μm.

Qualitative analysis for GFAP (Fig. 6) was based on the relative numbers of immunoreactive cells as well as the cellular morphology of the astrocyte. Resting astrocytes present a small cell body that extends thin highly-branched processes, whereas reactive astrocytes exhibit a large cell body with thick darkly stained processes. Resting astrocytes were found in the control animals throughout the cerebral cortex and hippocampus. However, in the hydrocephalic animals, there was a considerable increase in the number of reactive astrocytes throughout all six layers of the cerebral cortex and in the pyramidal cell layer of CA1-CA3 and the granular cell layer in the dentate gyrus in the hippocampus. There were no traces of astrocytes in the choroid plexus of either group.

### Quantitative RT-PCR

**Figure 7:**
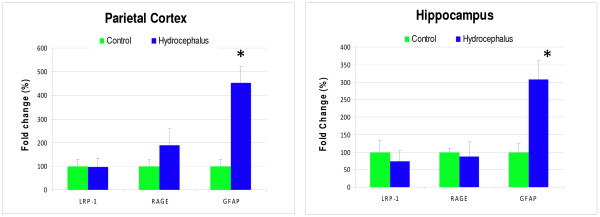
**Quantitative RT-PCR: The histograms represent quantitative results from the real time RT-PCR analysis**. The data indicate a significant increase in GFAP in both the parietal cortex and hippocampus of the hydrocephalic animals versus the age-matched controls. No significant differences were seen in LRP-1 and RAGE in the parietal cortex and hippocampus. The means are expressed as percent change versus control plus or minus the standard error of the mean. N= 5 for each group and statistical significance is defined as *P *< 0.05.

Quantitative real time RT-PCR data (Figure 7) showed that in both the parietal cortex and the hippocampus of the hydrocephalic animals, there were no statistical differences in LRP-1 and RAGE between hydrocephalic animals and age-matched sham controls (*P *> 0.05). Mean LRP-1 mRNA expression was 98 ± 15% of control in the parietal cortex of the hydrocephalic animals and 75 ± 8% of the control in the hippocampus. The mean RAGE mRNA expression exhibited an increase of 189 ± 58% in the parietal cortex of the hydrocephalic animals and 87 ± 12% of the control in the hippocampus. In contrast, GFAP in the parietal cortex and hippocampus revealed a significant increase (*P *< 0.05). In hydrocephalic animals, the mean GFAP mRNA expression showed an increase of 454 ± 140% and 308 ± 60% for the parietal cortex and hippocampus, respectively. Values are expressed as average comparative Ct values plus or minus the standard error of the mean.

### Amyloid-β protein and transporters in adult hydrocephalus

Immunohistochemical results for Aβ, LRP-1, and RAGE in adult hydrocephalic animals differed from those of the neonatal hydrocephalic animals. There was a substantial increase of Aβ and RAGE in the cortex (Fig. 3 and 5 respectively). LRP-1 in the adult rat was expressed around the blood vessels in the cortex (Fig. 4). On the other hand, RAGE (Fig. 5) and Aβ (Fig 3) were highly expressed around the blood vessels in the pyramidal cell layer, the granular cell layer of the dentate gyrus, and along the hippocampal fissure of the hippocampus. LRP-1 was minimally expressed in the hippocampus. The results for both adult and neonatal rats are summarized in Table 1.

**Table 1:** Summary of relative changes in expression of A-beta protein (Aβ), low-density lipoprotein receptor-related protein (LRP-1), receptor for advanced glycation end products (RAGE) and glial fibrillary acidic protein (GFAP) in brain tissue from control and hydrocephalic rats.

	Adult Hydro vs. Control IHC	Neonatal Hydro vs. Control IHC/qRT-PCR	Adult Control vs. Neonatal Control IHC	Adult Hydro vs. Neonatal Hydro IHC
Aβ	Increase	NC/NA	NC	Decrease
LRP-1	Decrease	NC/NC	Decrease	NC
RAGE	Increase	NC/NC	Decrease	Decrease
GFAP	Increase	Increase/Increase	NC	NC

IHC: immunohistochemistry; qRT-PCR: quantitative RT- PCR; NC- no change; NA-not applicable. Results from cortex and hippocampus have been combined.

## Discussion

In summary, intraventricular obstructive hydrocephalus induced in one day old rats created a severe state of ventriculomegaly during the 3-week post-induction period. It was apparent from both the MRI images and the histology sections that this severity caused dramatic thinning of the cortical mantle, loss of periventricular white matter, disorientation and shrinkage of cortical pyramidal cells, compression of the caudate-putamen and septum. For age-related comparisons, 21-day old hydrocephalic animals were compared to adult (6–12 month old) hydrocephalic animals; immunohistochemistry revealed levels of Aβ, RAGE, and LRP-1 that were substantially lower in the younger animals. Conversely, GFAP expression was elevated in both young and old hydrocephalic animals. When 21-day post-kaolin hydrocephalic animals were compared to age-matched controls, immunohistochemistry showed relatively small increases and decreases in individual proteins. The insignificance of these small changes is addressed more appropriately by qRT-PCR, which demonstrated no statistical differences in Aβ, LRP-1 and RAGE expression. In contrast, qRT-PCR also showed statistically significant increases in GFAP.

It is important to understand that when comparing neonatal to adult histology there are considerable differences in the appearance of the stains. When performing immunohistochemistry on the neonatal tissue for Aβ, LRP-1, and RAGE, we experienced great difficulty eliminating non-specific staining. This includes the dark brown outline around the neurons in figures 4 and 5, as well as the brown background of the cytoplasm in figures 3, 4, 5, 6. Rigorous attempts were made to identify the appropriate concentrations for each antibody to reduce the background stain but were relatively ineffective. Positively-labeled proteins can be characterized by small punctuate particles as well as diffuse profiles. Further immunoabsorption experiments with authentic (purified) epitopes could help overcome problems with non-specific labeling.

### Severity of hydrocephalus

The extent of ventriculomegaly and its effects on surrounding brain tissue have been described in other neonatal and juvenile models with expandable skulls[2,27,33-35], and the degree of tissue distortion is far greater than that seen in adult animals with fixed cranial sutures and non-expandable skulls[4,29,36-43]. Included in the latter category are the animals from studies by Klinge *et al*[15] and the human cases presented by Donahue *et al*[14] that demonstrated impaired amyloid protein clearance. Thus, our protein clearance findings may be different from previous reports because they occurred in a setting of more profound ventriculomegaly. However, because minimal labeling of transporters occurred in both control and hydrocephalic brains, it appears likely that the Aβ clearance system is not functional in neonatal rats. Thus, impairments cannot be detected in a transport system that does not have fully functional transporters. It is also well known that with severe hydrocephalus cerebral blood flow is decreased by at least 60%[44,45]. These decreases in cerebral blood flow could create ischemic conditions that dramatically affect Aβ transporters. Furthermore, since Aβ can enter the brain from the bloodstream[46], it is possible that less Aβ is delivered directly to the neuropil in an ischemic hydrocephalic brain. We saw very little evidence of RAGE expression and without the mechanism of transport into the brain, Aβ cannot accumulate. Finally, it is also possible that the severity of the hydrocephalus caused a decrease in neuronal amyloid precursor protein expression and that might have caused a reduction in Aβ production. It will be interesting to repeat the present study in animals with less severe hydrocephalus.

### Expression of transport proteins

Previously, Silverberg *et al*[32] and Donahue *et al*[14] have shown that there is an accumulation of Aβ in patients with adult onset hydrocephalus and Alzheimer's disease (AD) due to a deficiency of Aβ clearance mechanisms. Klinge *et al*[15], performed their studies in adult animals at 1 year of age, and showed a significant increase in Aβ around the blood vessels and the neuropil compared to non-hydrocephalic age-matched controls but no change was reported in the choroid plexus. The LRP-1 protein levels substantially decreased around the blood vessels of the cortex and hippocampus Klinge *et al *[15] and RAGE protein levels increased around the microvasculature of the cortex and hippocampus Donahue *et al*[14]. These levels were proportional to the severity of hydrocephalus.

RAGE expression in the brain is reported to increase during development when it interacts with amphoterin (HMBG-1) to induce neurite outgrowth and then decreases with maturation[47]. These changes have been observed in rats up to 12 months and then expression subsequently increases dramatically during aging. Thus RAGE is very age dependent in its expression.

In contrast, we did not observe Aβ in the cerebral cortex and hippocampus of our saline-control or hydrocephalic 21-day post-kaolin animals. LRP-1 was increased slightly in the cerebral cortex but decreased slightly in the hippocampus of hydrocephalic animals. RAGE however, showed opposite results in that it was decreased slightly in the cortex and there was no change seen in the hippocampus between the saline and hydrocephalic animals. This pattern differs from what has been described in the adult animal studies.

### Age-related changes

The most plausible explanation for the differences between our data and the findings of Silverberg, Klinge, and colleagues[15,48] is the age of the animals. In the adult studies, the animals were at least 1 year old, whereas our animals were only 3 weeks old. This age differential becomes important when one considers the maturation of protein clearance mechanisms. In the mature brain, Aβ is produced constantly, but is cleared rapidly in a normal working system where interstitial fluid concentration remains about 10–12 nM. The system is a bidirectional concentration-dependent flux across the blood brain barrier (BBB). When that system is intact, Aβ will not accumulate even if the CSF pathway is compromised. It is only when the BBB transporters are altered with age or disease that Aβ accumulates. Microvascular LRP-1 expression must be reduced and RAGE expression increased in order for Aβ to build up in the brain parenchyma. Silverberg *et al *have also observed very little Aβ accumulation in 3 month old rats (personal communication). Likewise, very little Aβ accumulation has been noted in young dogs[49]. Therefore, our results in immature animals are consistent with preliminary data from other labs.

The age differential is also important because the effects of hydrocephalus may be very different in immature brains compared to adults. The postnatal period from one day to 2 weeks is a watershed period for CSF formation (hydrodynamics)[50] by choroid plexus and re-absorptive openings in the arachnoid. Consequently, there are many changing endogenous variables throughout our experimental period. The CSF sink action[51], which plays a prominent role in aging rats, hardly exists in neonatal rats but it increases progressively from 0–3 days post-partum. Thus, kaolin injection resulting in ventriculomegaly just after birth could alter the normal development of CSF pathways and fluid kinetics.

Finally, it is possible that since plasma Aβ concentration is lower in infants than in adults there is a smaller concentration gradient for Aβ to diffuse from plasma to the brain. Furthermore, degradative pathways for Aβ may be upregulated in immature brains. For example, neprilysin and insulin degrading enzyme contribute to Aβ clearance in adult brains[52], it is not known if this pathway plays a more prominent role in developing brains.

The positive Aβ and LRP-1 staining for choroid plexus is interesting and suggests that the plexus is removing Aβ from CSF of hydrocephalic animals[13], which could explain the lack of Aβ staining in the hydrocephalic brain.

### GFAP expression

Gliosis is commonly found throughout the brain in animals with severe hydrocephalus[2,27]. Increases in GFAP and Aβ are commonly found together in adult brains with neurological deficits[17-24]. Qiao *et al*. [25], Funato *et al*[21] and Sasaki *et al*[53] state that there is a possibility that astrocytes engulf Aβ diffused plaques and attempt to degrade them in lysosomes in the aged brain. Qiao *et al*[25] found that neuroinflammation can accelerate amyloid deposition in mice and Nagele *et al*[24] suggest that Aβ-burdened neurons and astrocytes appear to make a major contribution to observed amyloid plaques in the brain. These studies point out that where there is an accumulation of reactive astrocytes in an aged brain one will find an accumulation of Aβ. In contrast, our hydrocephalic rats exhibited large amounts of reactive astrocytes but no appearance of Aβ. Therefore, we cannot make the same correlation for our younger animals. Our findings suggest that reactive astrocytosis may not be directly involved in protein clearance, or that other factors such as neuronal damage, ischemia, edema or intraparenchymal pressure may trigger an independent response in astrocytes.

## Conclusion

In neonatal hydrocephalic animals, immunohistochemical staining demonstrated levels of Aβ, RAGE, and LRP-1 that were substantially lower than those seen in adult hydrocephalic animals, while GFAP levels were elevated in both groups. Immunohistochemistry and qRT-PCR in neonatal rats revealed no significant change in Aβ, LRP-1 and RAGE between the hydrocephalic animals and age-matched controls. However, statistically significant increases in GFAP gene expression did occur in the hydrocephalic animals.

## Competing interests

The authors declare that they have no competing interests.

## Authors' contributions

KED participated in coordinating and designing the study, carried out the surgical and immunohistochemical procedures, and analyzed the data. JF carried out the molecular studies and performed the statistical analysis. OA and EH provided the MRI facilities and performed and interpreted the MRI scans. PMK, GDS and CEJ participated in framing the hypothesis, critiqued the final manuscript and provided comparative data. Additionally, PMK participated in immunohistochemistry procedures. JPM supervised the experiments, participated in the coordination and design of the study, obtained funding for the project, and assisted in analyzing the data and writing the manuscript. All authors have read and approved the final version of the manuscript.

## References

[B1] Rekate HL, Trimurti D, Nadkarni MCh, Wallace D (2008). The importance of the cortical subarachnoid space in understanding hydrocephalus. J Neurosurg Pediatrics.

[B2] Khan OH, Enno TL, Del Bigio MR (2006). Brain damage in neonatal rats following kaolin induction of hydrocephalus. Exp Neurol.

[B3] Del Bigio MR (2001). Pathophysiologic consequences of hydrocephalus. Neurosurg Clin N Am.

[B4] Del Bigio MR, McAllister JP, Choux M, DiRocco R, Hockley AD, Walker ML (1999). Pathophysiology of Hydrocephalus. Pediatric Neurosurgery.

[B5] McAllister JP, Chovan P (1998). Neonatal hydrocephalus. Mechanisms and consequences. Neurosurg Clin N Am.

[B6] Persson EK, Hagberg G, Uvebrant P (2006). Disabilities in Children with Hydrocephalus – A Population-Based Study of Children Aged Between Four and Twelve Years. Neuropediatrics.

[B7] Silverberg GD (2004). Normal pressure hydrocephalus (NPH): ischaemia, CSF stagnation or both. Brain.

[B8] Abbott NJ (2004). Evidence for bulk flow of brain interstitial fluid: significance for physiology and pathology. Neurochem Int.

[B9] Ghersi-Egea JF, Gorevic PD, Ghiso J, Frangione B, Patlak CS, Fenstermacher JD (1996). Fate of cerebrospinal fluid-borne amyloid beta-peptide: rapid clearance into blood and appreciable accumulation by cerebral arteries. J Neurochem.

[B10] Kapaki EN, Paraskevas GP, Tzerakis NG, Sfagos C, Seretis A, Kararizou E, Vassilopoulos D (2007). Cerebrospinal fluid tau, phospho-tau181 and beta-amyloid1-42 in idiopathic normal pressure hydrocephalus: a discrimination from Alzheimer's disease. Eur J Neurol.

[B11] Lins H, Wichart I, Bancher C, Wallesch CW, Jellinger KA, Rosler N (2004). Immunoreactivities of amyloid beta peptide((1–42)) and total tau protein in lumbar cerebrospinal fluid of patients with normal pressure hydrocephalus. J Neural Transm.

[B12] Weller RO, Nicoll JA (2003). Cerebral amyloid angiopathy: pathogenesis and effects on the ageing and Alzheimer brain. Neurol Res.

[B13] Johanson CE, Flaherty S, Messier A, Duncan JI, Silverberg G (2006). Expression of the beta-amyloid transporter, LRP-1, in aging choroid plexus: implications for the CSF-brain system in NPH and Alzheimer's disease. Cerebrospinal Fluid Research.

[B14] Donahue JE, Flaherty SL, Johanson CE, Duncan JA, Silverberg GD, Miller MC, Tavares R, Yang W, Wu Q, Sabo E, Hovanesian V, Stopa EG (2006). RAGE, LRP-1, and amyloid-beta protein in Alzheimer's disease. Acta Neuropathol.

[B15] Klinge PM, Samii A, Niescken S, Brinker T, Silverberg GD (2006). Brain amyloid accumulates in aged rats with kaolin-induced hydrocephalus. NeuroReport.

[B16] Gloeckner SF, Meyne F, Wagner F, Heinemann U, Krasnianski A, Meissner B, Zerr I (2008). Quantitative analysis of transthyretin, tau and amyloid-beta in patients with dementia. J Alzheimers Dis.

[B17] Albrechtsen M, Sorensen PS, Gjerris F, Bock E (1985). High cerebrospinal fluid concentration of glial fibrillary acidic protein (GFAP) in patients with normal pressure hydrocephalus. J Neurol Sci.

[B18] Pike CJ, Vaughan PJ, Cunningham DD, Cotman CW (1996). Thrombin attenuates neuronal cell death and modulates astrocyte reactivity induced by β-amyloid in vitro. J Neurochem.

[B19] Tullberg M, Rosengren L, Blomsterwall E, Karlsson JE, Wikkelso C (1998). CSF neurofilament and glial fibrillary acidic protein in normal pressure hydrocephalus. Neurology.

[B20] Dudal S, Krzywkowski P, Paquette J, Morissette C, Lacombe D, Tremblay P, Gervais F (2004). Inflammation occurs early during the Abeta deposition process in TgCRND8 mice. Neurobiol Aging.

[B21] Funato H, Yoshimura M, Yamazaki T, Saido TC, Ito Y, Yokofujita J, Okeda R, Ihara Y (1998). Astrocytes containing amyloid beta-protein (Abeta)-positive granules are associated with Abeta40-positive diffuse plaques in the aged human brain. Am J Pathol.

[B22] Mandybur TI, Chuirazzi CC (1990). Astrocytes and the plaques of Alzheimer's disease. Neurology.

[B23] Nagele RG, Wegiel J, Venkataraman V, Imaki H, Wang KC, Wegiel J (2004). Contribution of glial cells to the development of amyloid plaques in Alzheimer's disease. Neurobiol Aging.

[B24] Nagele RG, D'Andrea MR, Lee H, Venkataraman V, Wang HY (2003). Astrocytes accumulate A beta 42 and give rise to astrocytic amyloid plaques in Alzheimer disease brains. Brain Res.

[B25] Qiao X, Cummins DJ, Paul SM (2001). Neuroinflammation-induced acceleration of amyloid deposition in the APPV717F transgenic mouse. Eur J Neurosci.

[B26] Yu WH, Go L, Guinn BA, Fraser PE, Westaway D, McLaurin J (2002). Phenotypic and functional changes in glial cells as a function of age. Neurobiol Aging.

[B27] Miller JM, McAllister JP (2007). Reduction of astrogliosis and microgliosis by cerebrospinal fluid shunting in experimental hydrocephalus. Cerebrospinal Fluid Res.

[B28] Del Bigio MR (2004). Cellular damage and prevention in childhood hydrocephalus. Brain Pathol.

[B29] Li J, McAllister JP, Shen Y, Wagshul ME, Miller JM, Egnor MR, Johnston MG, Haake EM, Walker ML (2008). Communicating hydrocephalus in adult rats with kaolin obstruction of the basal cisterns or the cortical subarachnoid space. Exp Neurol.

[B30] McAllister JP, Maugans TA, Shah MV, Truex RC (1985). Neuronal effects of experimentally induced hydrocephalus in newborn rats. J Neurosurg.

[B31] Schmittgen TD, Livak KJ (2008). Analyzing real-time PCR data by the comparative C(T) method. Nat Protoc.

[B32] Silverberg GD, Mayo M, Saul T, Rubenstein E, McGuire D (2003). Alzheimer's disease, normal-pressure hydrocephalus, and senescent changes in CSF circulatory physiology: a hypothesis. The Lancet Neurology.

[B33] Del Bigio MR, Crook CR, Buist R (1997). Magnetic resonance imaging and behavioral analysis of immature rats with kaolin-induced hydrocephalus: pre- and postshunting observations. Exp Neurol.

[B34] Del Bigio MR, Bruni JE (1991). Silicone oil-induced hydrocephalus in the rabbit. Childs Nerv Syst.

[B35] Shoesmith CL, Buist R, Del Bigio MR (2000). Magnetic resonance imaging study of extracellular fluid tracer movement in brains of immature rats with hydrocephalus. Neurol Res.

[B36] Del Bigio MR, Bruni JE, Vriend JP (1998). Monoamine neurotransmitters and their metabolites in the mature rabbit brain following induction of hydrocephalus. Neurochem Res.

[B37] Johanson C, Del Bigio MR, Kinsman S, Miyan J, Pattisapu JV, Robinson M, Jones HC (2001). New models for analysing hydrocephalus and disorders of CSF volume transmission. Br J Neurosurg.

[B38] Shapiro K, Takei F, Fried A, Kohn I (1985). Experimental feline hydrocephalus. The role of biomechanical changes in ventricular enlargement in cats. J Neurosurg.

[B39] Fried A, Shapiro K, Takei F, Kohn I (1987). A laboratory model of shunt-dependent hydrocephalus. Development and biomechanical characterization. J Neurosurg.

[B40] Johnson MJ, Ayzman I, Wood AS, Tkach JA, Klauschie J, Skarupa DJ, McAllister JP, Luciano MG (1999). Development and characterization of an adult model of obstructive hydrocephalus. J Neurosci Methods.

[B41] Hochwald GM, Sahar A, Sadik AR, Ransohoff J (1969). Cerebrospinal fluid production and histological observations in animals with experimental obstructive hydrocephalus. Exp Neurol.

[B42] Weller RO, Wisniewski H, Shulman K, Terry RD (1971). Experimental hydrocephalus in young dogs: histological and ultrastructural study of the brain tissue damage. J Neuropathol Exp Neurol.

[B43] Aoyama Y, Kinoshita Y, Yokota A, Hamada T (2006). Neuronal damage in hydrocephalus and its restoration by shunt insertion in experimental hydrocephalus: a study involving the neurofilament-immunostaining method. J Neurosurg.

[B44] Jones HC, Richards HK, Bucknall RM, Pickard JD (1993). Local cerebral blood flow in rats with congenital hydrocephalus. J Cereb Blood Flow Metab.

[B45] da Silva MC, Michowicz S, Drake JM, Chumas PD, Tuor UI (1995). Reduced local cerebral blood flow in periventricular white matter in experimental neonatal hydrocephalus-restoration with CSF shunting. J Cereb Blood Flow Metab.

[B46] Deane R, Du YS, Submamaryan RK, Larue B, Jovanovic S, Hogg E, Welch D, Manness L, Lin C, Yu J, Zhu H, Ghiso J, Frangione B, Stern A, Schmidt AM, Armstrong DL, Arnold B, Liliensiek B, Nawroth P, Hofman F, Kindy M, Stern D, Zlokovic B (2003). RAGE mediates amyloid-beta peptide transport across the blood-brain barrier and accumulation in brain. Nat Med.

[B47] Leclerc E, Fritz G, Weibel M, Heizmann CW, Galichet A (2007). S100B and S100A6 differentially modulate cell survival by interacting with distinct RAGE (receptor for advanced glycation end products) immunoglobulin domains. J Biol Chem.

[B48] Klinge PM, Samii A, Muhlendyck A, Visnyei K, Meyer GJ, Walter GF, Silverberg GD, Brinker T (2003). Cerebral hypoperfusion and delayed hippocampal response after induction of adult kaolin hydrocephalus. Stroke.

[B49] Cavaglia M, Dombrowski SM, Drazba J, Vasanji A, Bokesch PM, Janigro D (2001). Regional variation in brain capillary density and vascular response to ischemia. Brain Res.

[B50] Johanson CE, Parandoosh Z, Dyas ML (1992). Maturational differences in acetazolamide-altered pH and HCO3 of choroid plexus, cerebrospinal fluid, and brain. Am J Physiol.

[B51] Parandoosh Z, Johanson CE (1982). Ontogeny of blood-brain barrier permeability to, and cerebrospinal fluid sink action on, [14C]urea. Am J Physiol.

[B52] Iwata N, Mizukami H, Shirotani K, Takaki Y, Muramatsu S, Lu B, Gerard NP, Gerard C, Ozawa K, Saido TC (2004). Presynaptic localization of neprilysin contributes to efficient clearance of amyloid-beta peptide in mouse brain. J Neurosci.

[B53] Sasaki N, Toki S, Chowei H, Saito T, Nakano N, Hayashi Y, Takeuchi M, Makita Z (2001). Immunohistochemical distribution of the receptor for advanced glycation end products in neurons and astrocytes in Alzheimer's disease. Brain Res.

